# Promising effects of topical sunflower seed oil, rich in linoleic acid, in infants with severe acute malnutrition

**DOI:** 10.1038/s41390-026-04907-7

**Published:** 2026-03-09

**Authors:** Philip C. Calder

**Affiliations:** 1 School of Human Development and Health, Faculty of Medicine, University of Southampton, Southampton, UK; 2 NIHR Southampton Biomedical Research Centre, University Hospital Southampton NHS Foundation Trust and University of Southampton, Southampton, UK

Severe acute malnutrition in children remains a major public health issue in parts of Asia and Africa. Compromized skin barrier function, seen as scaling, plaques and ulcerations, is a feature of severe acute malnutrition and increases risk of infection, since pathogens can more easily gain entry to the body.^1^ In this issue of *Pediatric Research*, Shahunja et al. report new findings from a randomized, controlled trial of topical sunflower seed oil (SSO) in young Bangladeshi children with severe acute malnutrition and receiving nutritional rehabilitation.^2^ The children were aged 2–24 months and received 3 g SSO per kg body weight three times a day for 10 days. The active component of SSO is likely the omega-6 fatty acid linoleic acid which comprises about 60% of the fatty acids present. Thus, these children were receiving about 1.8 g linoleic acid topically three times a day. Linoleic acid is an essential fatty acid produced in plants and often stored in the seeds. Hence many seeds, nuts and plant seed oils are a rich source of linoleic acid, as are foods made from or containing these sources. Linoleic acid is an important constituent of ceramides, particularly those found in the skin.^3^ Hence, essential fatty acid deficiency results in breakdown of skin integrity and inability to prevent transdermal water loss. Thus, there is a rationale in providing linoleic acid topically in malnourished infants alongside standard oral nutritional repletion. In support of this, topical SSO has been reported to improve skin barrier function, reduce transdermal water loss, reduce infections and improve weight gain in preterm infants.^4–8^ Linoleic acid has other important roles beyond the skin. For example, it regulates hepatic lipid metabolism, such that it lowers blood total and low-density lipoprotein cholesterol concentrations,^9^ it is metabolized to oxidized derivatives (hydroxyoctadecadienoic acids) which are involved in inflammation^10^ and it is a substrate for synthesis of arachidonic acid the main precursor for eicosanoid metabolites.^11^ Despite its cholesterol-lowering effects, there has been some debate around the role of linoleic acid and seed oils rich in linoleic acid in human health.^12^ Nevertheless, data from large cohort studies and from trials in adults confirm that linoleic acid is associated with reduced risk of both coronary heart disease and type-2 diabetes, especially when it replaces saturated fatty acids in the diet.^13–15^ However, health-related effects of linoleic acid likely extend beyond cardiometabolic disease into immunity and inflammation and the microbiome.

In this new publication, Shahunja et al.^2^ explore effects of topical SSO on systemic immune markers, the rationale being that children with severe acute malnutrition have weakened immunity and increased risk of infections.^16,17^ While nutritional rehabilitation itself is reported to help reverse that situation, in previous reports from this trial, topical SSO in combination with nutritional rehabilitation had effects beyond the rehabilitation alone.^18,19^ For example, topical SSO increased weight gain (the primary outcome of this trial) beyond that seen with nutritional rehabilitation alone, although the difference between groups did not reach statistical significance.^18^ However, SSO significantly decreased the rate of nosocomial infections in children aged 6–24 months (though not in those aged 2– 6 months), improved skin quality, decreased transdermal water loss^18^ and altered the skin, though not fecal, microbiota with increased bacterial diversity being seen.^19^ Furthermore, in the children aged 2–6 months, SSO decreased blood C-reactive protein concentration.^18^ In the latest publication from this trial, Shahunja et al.^2^ report no effects of topical SSO on serum cytokine (tumor necrosis factor, interleukins 8 and 10) concentrations; the concentrations of most cytokines measured were below the detectable range. This finding is interpreted by the authors as indicating no effect of topical SSO on systemic immunity. This may be the case, but there are a multitude of immune biomarkers beyond cytokines, including immune cell numbers and phenotypes, specific immune cell functional responses and immunoglobulin concentrations. Since none of these have been measured it seems premature to completely dismiss an effect of topical SSO on systemic immunity, particularly in light of the effect on serum C-reactive protein in the younger children. Nevertheless, the rather modest systemic effects and the lack of effect on gut microbiota^2,18,19^ alongside the improved skin quality, decreased transdermal water loss and altered skin microbiota^18,19^ strongly suggest that topical SSO has local cutaneous effects. These may relate to a normalized skin ceramide profile, although little is known about skin ceramides in severe acute malnutrition in children, and direct effects of linoleic acid or its metabolites on keratinocytes, fibroblasts and immune cells^20–22^ within the skin.

The findings presented by Shahunja et al.^2^ and in the earlier publications from this trial^18,19^ are interesting and point to a low-cost intervention that might be clinically meaningful in severe acute malnutrition in young children in low resource settings. Limitations of the research include the relatively small sample size (*n* = 106 per group), the short duration of the intervention (10 days) and the unblinded study design. Strengths include the high retention of participants (over 90%) and the high compliance to the intervention because it was delivered by health professionals during the period of hospitalization. These findings call for a larger and longer double blind randomized controlled trial of SSO that should include a more detailed assessment of cutaneous and systemic outcomes alongside the clinical assessments.
